# The root meristem is shaped by brassinosteroid control of cell geometry

**DOI:** 10.1038/s41477-021-01014-9

**Published:** 2021-11-15

**Authors:** Y. Fridman, S. Strauss, G. Horev, M. Ackerman-Lavert, A. Reiner-Benaim, B. Lane, R. S. Smith, S. Savaldi-Goldstein

**Affiliations:** 1 Faculty of Biology, Technion-Israel Institute of Technology, Haifa, Israel; 2 Department of Comparative Development and Genetics, Max Planck Institute for Plant Breeding Research, Cologne, Germany; 3 Lorey I. Lokey Interdisciplinary Center for Life Sciences and Engineering, Technion – Israel Institute of Technology, Haifa, Israel; 4 Clinical Epidemiology Unit, Rambam Health Care Campus, Haifa, Israel; 5 Department of Computational and Systems Biology, John Innes Centre, Norwich, UK

**Keywords:** Plant morphogenesis, Brassinosteroid, Root apical meristem

## Abstract

Growth extent and direction determine cell and whole-organ architecture. How they are spatio-temporally modulated to control size and shape is not well known. Here we tackled this question by studying the effect of brassinosteroid (BR) signalling on the structure of the root meristem. Quantification of the three-dimensional geometry of thousands of individual meristematic cells across different tissue types showed that the modulation of BR signalling yields distinct changes in growth rate and anisotropy, which affects the time that cells spend in the meristem and has a strong impact on the final root form. By contrast, the hormone effect on cell volume was minor, establishing cell volume as invariant to the effect of BR. Thus, BR has the highest effect on cell shape and growth anisotropy, regulating the overall longitudinal and radial growth of the meristem, while maintaining a coherent distribution of cell sizes. Moving from single-cell quantification to the whole organ, we developed a computational model of radial growth. The simulation demonstrates how differential BR-regulated growth between the inner and outer tissues shapes the meristem and thus explains the non-intuitive outcomes of tissue-specific perturbation of BR signalling. The combined experimental data and simulation suggest that the inner and outer tissues have distinct but coordinated roles in growth regulation.

## Abstract (web summary)

What is the effect of hormones on cell shape and growth? In this study, morphometric, genetic and computer modelling analyses are combined to show how brassinosteroids control overall root meristem geometry and development.

## Main

**Fig. 1:**
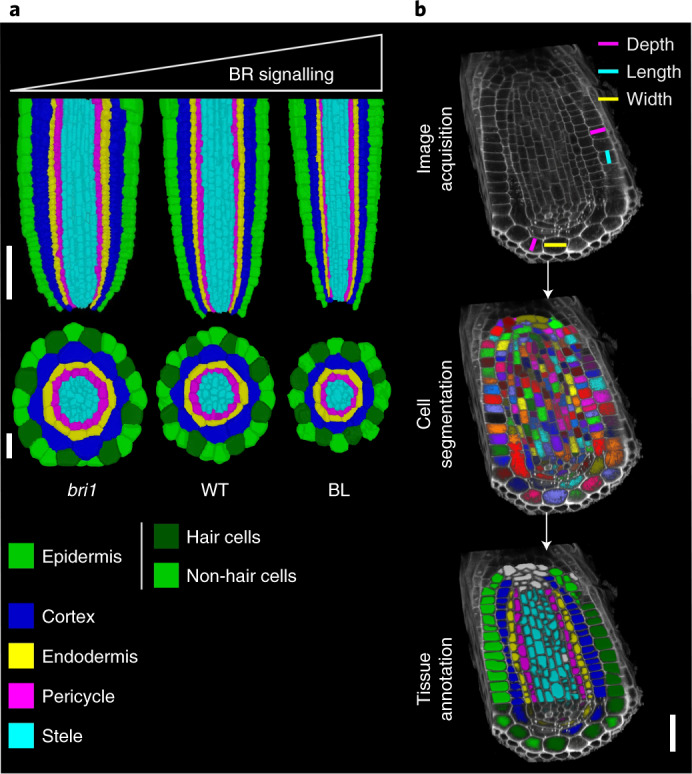
Root meristem morphology and segmentation scheme. **a**, Confocal images presented as longitudinal (top) and radial (bottom) cross sections of the root tip of a seven-day-old seedling, showing morphological differences between *bri1*, WT and WT grown in the presence of BL for four days. The cells underwent membrane-based segmentation and were classified into different tissues, as shown by different pseudo-colours. Note the decreasing root diameter with increasing BR signalling. Longitudinal scale bar, 50 μm; radial scale bar, 20 μm. **b**, Summary of key steps to obtain tissue-specific 3D geometric parameters. Volumetric cell geometries and their positioning in root cells are marked. Scale bar, 20 μm.

Plant morphogenesis is determined by the rate of growth (cell expansion and cell division) and its directionality (anisotropy)^1^. Growth rates are governed by hormonal signalling, the decoding of which depends on hormone levels and on the tissue and cell type in which it occurs. How hormonal signalling coordinates growth anisotropy remains unclear. The primary root meristem is composed of concentric tissue files surrounding the innermost stele cells (Fig. 1). In the longitudinal axis, stem cell daughters undergo a series of anticlinal divisions in their corresponding tissue file before they begin to rapidly elongate in the elongation zone^2^. The root meristem also expands in width (in the radial axis) by a series of periclinal divisions that increase the number of procambium cells in the stele and by tangential divisions that add additional cell files to selected tissues, involving interwoven transcriptional factors and hormonal signals^3,4^.

As cells grow in a tissue context, they are subjected to mechanical feedbacks^5–7^, which control whole-organ shape. Kinematics and additional quantifications of root growth parameters have been used to assess relative changes in growth rates among genotypes and treatments^8^. However, the analysis of the effect of hormones on three-dimensional (3D) root growth on a cellular scale is just emerging^9^, and data on the accompanying changes in cell volume alongside growth anisotropy are lacking.

Brassinosteroid (BR) signalling regulates cell length and meristematic cell counts in the root in both the longitudinal and radial axes^10,11^. The signalling is initiated upon binding of the hormone to its cell surface receptor, BRI1, through a regulatory sequence involving the inactivation of the GSK3 kinase BIN2, which plays a major inhibitory role by phosphorylating and thereby inhibiting the activity of key downstream transcription factors belonging to the BES1/BZR1 family^12^. High BR levels limit the number of dividing cells in the longitudinal and radial axes, promote early exit from the meristem, and increase cell length^13–15^. The *bri1* mutant has a short meristem with reduced cell cycle activity^13,16^ and an increased number of cells in the radial axis^14,17^. Morphologically, these meristems have longer cells arranged within a narrow structure (high BR) or have shorter cells arranged within a wider structure (low BR). However, BR signalling at the cellular scale has non-intuitive effects on the whole-root meristem structure^10,18^. Specifically, limiting BRI1 expression to the outer tissues promotes meristem length and restricts the meristem width, while limiting BRI1 to the stele has the opposite effect, resulting in a meristem structure that is wider than that of *bri1* (refs. ^16,17,18^). Similarly, the inhibition of BR signalling via the expression of the dominant-active version of BIN2 in only the outer and the inner tissues yielded wider and narrower meristems than the wild type (WT), respectively^19^, highlighting the essential role of the outer tissues in promoting longitudinal growth and restricting radial growth. It has also been proposed that BR signalling regulation of BR biosynthesis genes in the inner stele tissue modulates BR levels that are perceived in the outer tissues, thus providing a mode of inter-tissue coordination^19^. In addition, BR intermediates appear to move within the meristem^20^. However, data on how BR signalling controls geometry on a cellular scale and how it integrates to the whole-organ scale, as with radial growth of the root meristem, is lacking.

A better understanding of morphogenesis and the regulatory signalling involved requires precise single-cell tools that quantify growth parameters in 3D at the cell scale^21–26^. However, their application is still scarce, and quantitative analysis of cellular growth rates in 3D (that is, volumetric qunatification within four-dimensional (4D) analysis) is rarely performed ^27^, mainly due to difficulties of segmenting microscopy images. Here, we quantified the geometry of meristematic cells in *Arabidopsis* roots with adequate, low and high BR signalling, using 3D and 4D analyses. We then integrated experimental data in a computational model of radial meristem growth and revealed how BR signalling shapes the meristem at the cell level and how tissue-specific constraints, modulated by BR, yield a coherent morphological output.

## BR signalling modifies cell shape but not volume

MorphoGraphX^28^ was deployed to precisely quantify the 3D geometry of meristematic cells in WT and BR-signalling-perturbed roots and to compare the length, width, depth, surface area and volume of various tissues (Fig. 1 and Supplementary Video 1). Virtually all cells of the root meristem of WT and *bri1* roots and of WT roots treated with the BR brassinolide (BL), hereafter referred to as ‘treatments’, were segmented (except lateral root cap (LRC) and stele cells inner to the pericycle). In total, we accurately segmented and analysed 8,849 cells (Supplementary Table 1).

**Fig. 2:**
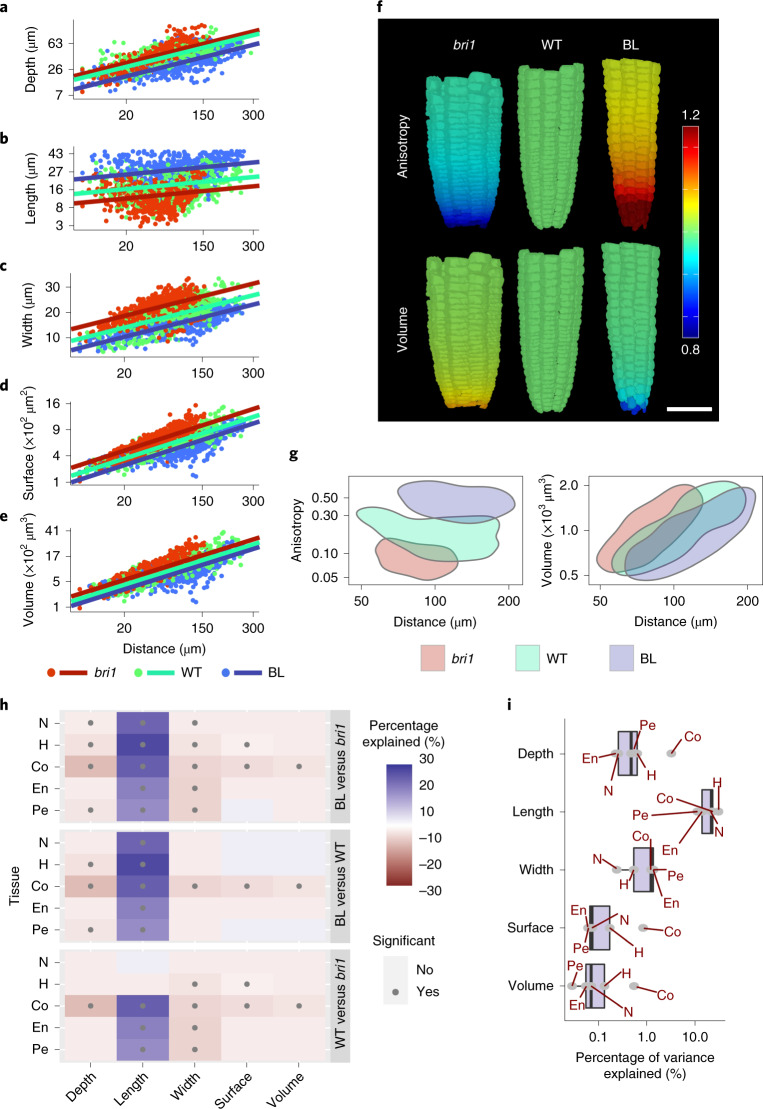
BR signalling has a higher effect on cell shape and anisotropy than on cell volume across meristematic tissues. **a**–**e**, Robust differences in geometric parameters of individual meristematic cells as captured by a mixed-model ANOVA (see the text for the details). Shown here are the effects of the distance from the QC (*x* axis, square-root-transformed) and BR signalling strength (differing among *bri1*, WT and BL-treated WT roots) on cell depth (log-transformed) (**a**), length (log-transformed) (**b**), width (**c**), surface area (square-root-transformed) (**d**) and volume (cube-root-transformed) (**e**) in the cortex. The axes are scaled according to the transformations. Corresponding analyses in other tissues are shown in Extended Data Fig. 2. See **h** for significant pairwise comparisons that result from this analysis. Note the opposite trend between roots with high and low BR signalling. Also note that all geometric parameters except length are higher in *bri1*. **f**,**g**, Comparison of anisotropy index (calculated as length^2^/(depth × width)) and volume between *bri1*, WT and BL-treated WT roots. In **f**, a display of the cortex tissue in representative segmented roots is shown, depicting relative differences in anisotropy index and volume (WT = 1) as a function of distance from the QC. In **g**, 2D kernel density plots of anisotropy index (left) and volume (cube-root-transformed, right) versus distance from the QC of WT, *bri1* and BL-treated WT root cells are shown. Note that BL-treated cells have significantly higher anisotropy, while *bri1* cells have significantly lower anisotropy. In contrast, the two groups of cells showed similar volume values. *P* < 0.05; mixed model. **h**, BR signalling has a higher effect on cell length, depth and width parameters than on cell volume across tissues. The heat map presents the percentage of variance explained by BR. Shown are all pairwise comparisons organized in three blocks (WT versus *bri1*, WT roots treated with BL versus WT and WT roots treated with BL versus *bri1*), for five geometric parameters in five root cell types and tissues. Blue indicates that the first treatment in the comparison has a higher value. Red indicates that the first treatment in the comparison has a lower value. The higher the opacity, the higher the percentage of variance explained. The dots indicate significance (adjusted *P* < 0.05; two-step adaptive correction; Methods). Note the significant, robust, opposing differences between *bri1* and BL-treated samples in length, depth and width across tissues, while cell volume remained largely stable. **i**, Box plot summarizing the effect of treatment on geometric parameters in terms of percent variance explained by BR signalling in a given tissue (grey dots). The plot shows the interquartile range as the left and right boundaries of the box, the median as an internal vertical line, and the maximum and minimum values as whiskers. For all panels, *n* = 4 roots for WT, 4 roots for *bri1* and 3 roots for BL. N, non-hair cells of the epidermis (*n* = 374 cells for WT, 376 cells for *bri1* and 280 cells for BL); H, hair cells of the epidermis (*n* = 288 cells for WT, 322 cells for *bri1* and 460 cells for BL); Co, cortex (*n* = 615 cells for WT, 532 cells for *bri1* and 462 cells for BL); En, endodermis (*n* = 445 cells for WT, 389 cells for *bri1* and 622 cells for BL); Pe, pericycle (*n* = 633 cells for WT, 749 cells for *bri1* and 833 cells for BL).

**Extended Data Fig. 1:**
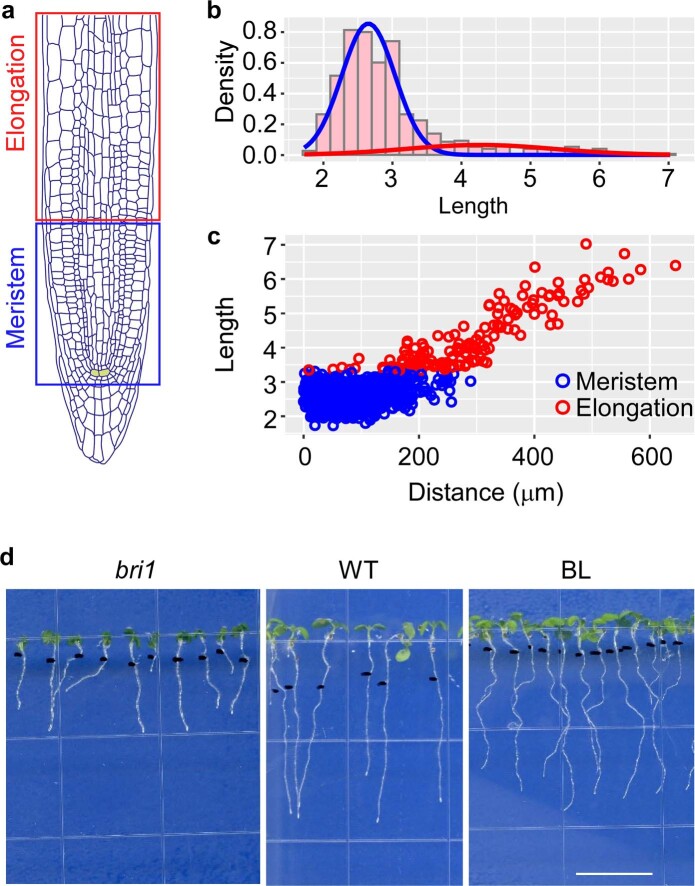
Algorithm-based labeling of meristematic cells and elongating cells. **a**, Schematic presentation of the meristem and elongation zone. The quiescent center (QC) cells are marked in yellow. **b**, Gaussian mixture model of cell length captures two populations of relatively short (blue gaussian) and long (red gaussian) cells. **c**, Scatter plot of the cells in the upper panel, depicting cell length vs. distance from the QC. Each dot represents a single cell of the meristem (blue) and elongation (red) zone. An example is shown for cells of WT cortex tissue. **d**, Morphology of 7-day-old seedlings of *bri1*, WT and WT treated with BL for 4 days. Scale bar =10 mm.

**Extended Data Fig. 2:**
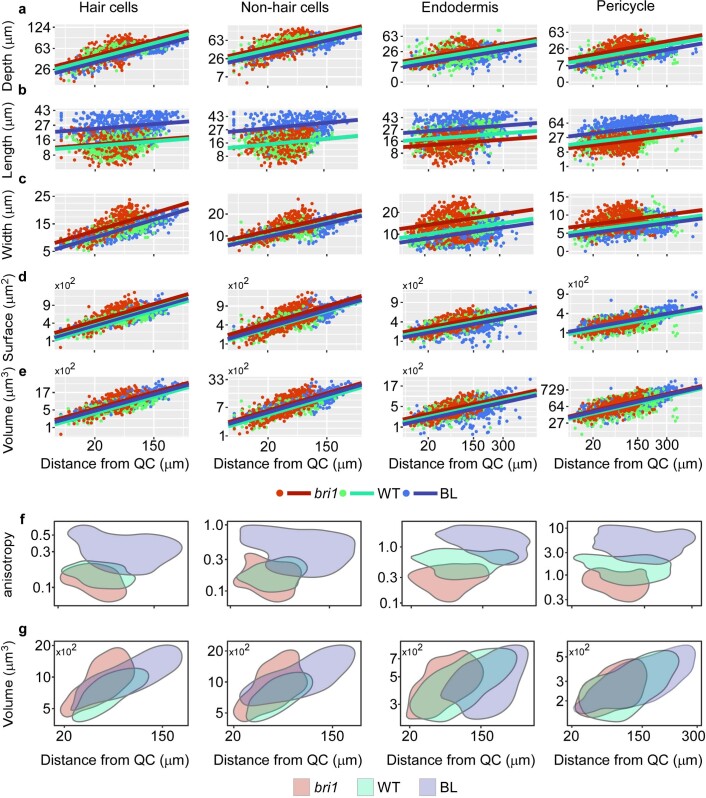
Low and high BR signaling show opposing trends in geometry parameters across meristematic tissues. **a-e**, Differences in single-cell geometric parameters. Shown are the effects of the distance from QC (x axis, square root-transformed) and roots with distinct BR signaling strength (i.e., WT, *bri1* and BL treated roots) on depth (log-transformed) (**a**), length (log-transformed) (**b**), width (**c**), surface area (square root-transformed) (**d**) and volume (cube root-transformed) (**e**) in epidermis (hair- and non-hair cells), endodermis and pericycle cells. Axes are scaled according to the transformations. Note the opposite trend between roots with high and low BR signaling. **f,g,** Two-dimensional kernel density plots of anisotropy index (**f**) and volume (cube root-transformed) (**g**) vs. distance from the quiescent center (QC), of WT, *bri1* and BL-treated WT root cells. All pair comparisons for anisotropy index are significantly different (except for H- and N- cells, *bri1* vs WT). p-value < 0.05; mixed model. For all panels: n= WT, 4 roots; *bri1*, 4 roots, and BL, 3 roots. Non-hair cells of the epidermis (n = WT, 374 cells; *bri1*, 376 cells; BL, 280); hair cells of the epidermis (n = WT, 288 cells; *bri1*, 322 cells; BL, 460 cells); endodermis, (n = WT, 445 cells; *bri1*, 389 cells; BL, 622 cells); pericycle (n = WT, 633 cells; *bri1*, 749 cells; BL, 833 cells).

**Extended Data Fig. 3:**
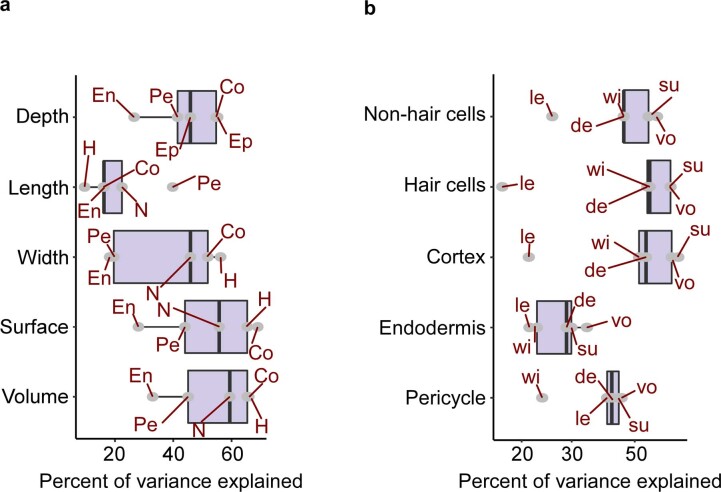
Percent variance of geometry parameters explained by distance from QC. **a-b**, Boxplots summarizing the percent variance explained by distance from QC, for all treatments, for each geometric parameter in a given tissue (gray dots). Boxes are grouped by geometric parameter (**a**) or by tissue (**b**). The plots show the interquartile range as the upper and lower boundaries of the box, the median as an internal vertical line and the maximum and minimum values as whiskers. The sample size used to derived statistics is as in Extended Data Fig. 2. Note that length is the geometric parameter and endodermis is the tissue with the least dependence on distance. Depth (de), length (le), width (wi), surface area (su), volume (vo).

To compare single cells of a similar developmental state, the cell population of the meristem zone was chosen for analysis (7,859 cells, Extended Data Fig. 1). To compare between treatments, mixed-model analysis of variance (ANOVA) (Methods) was used, as it sets a high benchmark of significance and therefore results in robust and replicable differences between treatments. Before we performed the mixed-model analysis, the different geometry parameters were transformed to achieve a proxy for a normal distribution (Supplementary Table 2). A corresponding transformation of a given geometry parameter was similarly applied for all tissues (epidermis hair cells, epidermis non-hair cells, cortex, endodermis and pericycle) in all three treatments. The mixed model was fitted for each parameter in each tissue, with treatment and distance from the quiescent centre (QC) as fixed effects and individual plants as a random effect. The statistical models were plotted as regression lines for the transformed parameter versus distance from the QC (Fig. 2a–e and Extended Data Fig. 2a–e). This revealed a gradual change along the meristem in cell volume and surface area, associated with an increase in cell depth and width. Cell length was the least affected by distance from the QC (Extended Data Fig. 3a). The data also showed an overall tendency for opposing effects of *bri1* and BL treatment on the geometric parameters (Fig. 2a–e and Extended Data Fig. 2a–e). BL-treated cells were longer, with reduced radial parameters (that is, depth and width), while *bri1* cells were shorter, with increased radial parameters. Next, cell geometry was expressed using an anisotropy index (that is, length^2^/(depth × width)), where relatively high values among cells indicate relatively more growth along the longitudinal axis than along the radial axes. We found that *bri1* cells had the lowest anisotropy index and BL-treated cells had the highest, with non-overlapping values between them and a small overlap with the WT (Fig. 2f,g and Extended Data Fig. 2f,g). Intriguingly, a parallel analysis of cell volume showed similar values in all treatments (Fig. 2f,g and Extended Data Fig. 2f,g).

To determine the magnitude of the BR signalling effect on each geometric parameter, the variance explained by treatment was quantified (Fig. 2h,i and Methods). Notably, when compared with untreated WT, BL-treated roots had significantly longer cells in all tissues and reduced cell depth in most of them. By contrast, *bri1* cells were shorter, with significantly higher width in most tissues. When comparing BL with *bri1*, the length, depth and width of cells significantly differed in all tissues (except endodermal depth), demonstrating a dose-dependent response to BR signalling on treatment with BL. However, in almost all pairwise comparisons, differences in volume remained non-significant between treatments (Fig. 2h). To summarize, we quantified and plotted what percentage of the variance is the result of treatment (that is, of BR signalling) for each geometry parameter in a given tissue (Fig. 2i). This demonstrated that volume and surface area were the least affected geometric parameters. Together, this quantitative single-cell geometry analysis demonstrated that BR signalling primarily promotes anisotropic growth. An apparent trade-off between length and depth/width, modulated by the intensity of the BR signalling, ensures cell volume conservation. This trade-off could also be the result of volume serving as a limiting factor.

## 3D time-lapse captures directional growth rate and geometry compensation

**Fig. 3:**
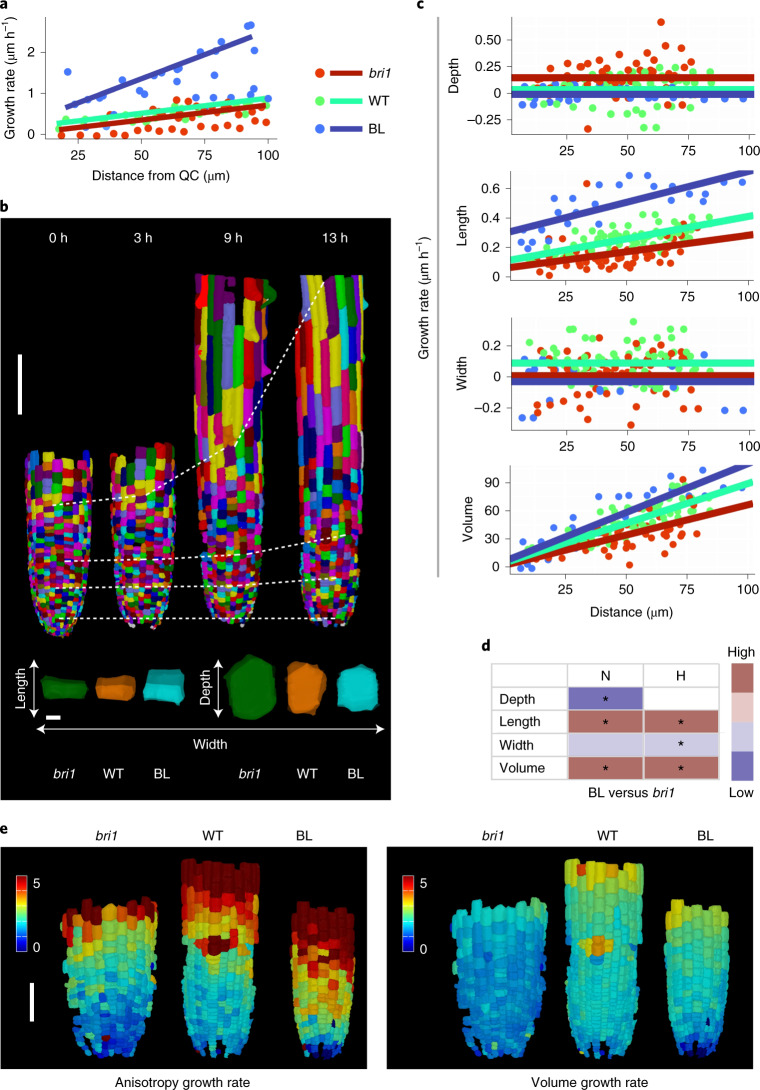
Time-lapse showing distinct growth rates in alternate directions, depending on BR signalling. **a**, Rate of epidermal cell displacement along the root meristem. Results are shown for WT, *bri1* and WT treated with BL. *n* = 4 roots for WT, 4 roots for *bri1* and 5 roots for BL. **b**, 3D segmentation of epidermal cells of WT roots, imaged at 0, 3, 9 and 13 h (top), and an overlay of single meristematic cells (bottom) at *t* = 0 (coloured with full opacity) and *t* = 10.5 h, 13 h and 12 h for *bri1*, WT and WT treated with BL, respectively (coloured with reduced opacity). Scale bars, 100 μm (top) and 5 μm (bottom). **c**, Single-cell growth in 4D of epidermal (non-hair) cells. Differences in the rate of cell growth (depth, length, width and volume) over the duration of imaging were modelled according to the position of the cells from the QC at the beginning of the imaging, in WT, *bri1* and WT treated with BL. Linear regressions were computed using analysis of covariance (ANCOVA). The numbers of individual non-hair cells quantified in the experiment are 130 for WT, 119 for *bri1* and 68 for BL (one root for each treatment). **d**, Differences in growth rates upon 4D analysis (as in **b** and Extended Data Fig. 4) for hair and non-hair epidermal cells of WT treated with BL samples and *bri1* samples, summarized as a heat map. Significant differences are marked by asterisks (adjusted *P* < 0.05; ANCOVA followed by contrast test; Methods). **e**, A display of anisotropy 4D index (left) and volume rate (right) of the corresponding meristematic cells in *bri1*, WT and WT treated with BL, as in **c**. Scale bar, 50 μm.

It remained unclear how these differences in cell shape are generated over time. More specifically, it remained to be determined whether they are a function of the time that cells spend in the meristem (the duration of growth), the rate of growth in a given axis or a combination of both. As a first step, we performed a kinematics study on two-dimensional (2D) images to quantify the rate of cell displacement along the root. To this end, the growth of epidermal cells along the meristem was imaged and monitored at 30-min intervals for a duration of 6 h (Fig. 3a and Supplementary Video 2). The analysis revealed a slow displacement of cells (several micrometres per hour) that increased with distance from the QC, within the region measured (100 μm). The relative change in cell displacement rate was lower for *bri1* cells and was much higher in the presence of BL (0.57-fold and 3-fold relative to the WT, respectively; Supplementary Table 3). Moreover, calculation of cell displacement rate at the end of the meristem (Methods and Supplementary Table 4) demonstrated that cells spend more time in the meristem in the absence of BR signalling and quickly exit it when BR levels are high.

**Extended Data Fig. 4:**
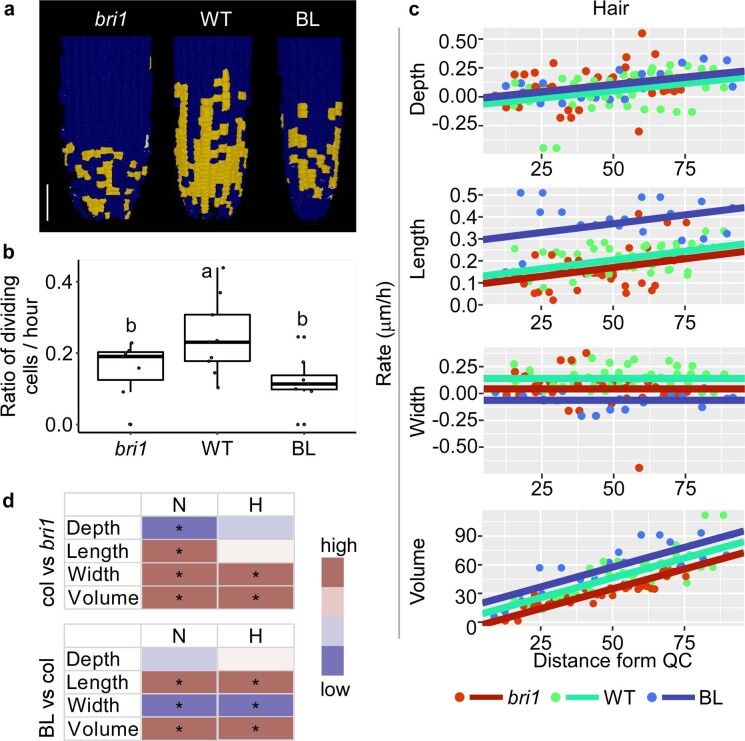
Cell division and growth of individual cells in 3D over time. **a**, Epidermal cells that underwent divisions in *bri1*, WT and WT treated with BL after 10.5 h, 13 h and 12 h of imaging, respectively, are marked in yellow, scale bar = 50 μm. **b**, The fraction of meristematic cells that underwent divisions is shown as the number of mother cells divided by total meristematic cells, normalized to imaging duration. Boxplot shows the interquartile range as the upper and lower boundaries of the box, the median as an internal horizontal line and the maximum and minimum values as whiskers. Each dot shows a ratio in a given epidermal cell file. Number of dividing cells (and number of meristematic cells) are 65 (223), 26 (157) and 23 (160) for WT, *bri1* and BL. Roots as in Fig. 3c. **c**, Differences in expansion rate (depth, length, width and volume) of hair-cells were plotted as a function of distance from the QC in WT, *bri1* and WT treated with BL, using analysis of covariance (ANCOVA). Number of individual hair cells quantified in the experiment are 76, 68 and 96 for WT, *bri1*, and BL, Roots as in Fig. 3c. **d**, Differences in growth rates upon 4D analysis as in (**c** and as in Fig. 3) for hair- and non-hair epidermal cells of WT treated with BL and *bri1* samples, summarized as a heatmap. Significant differences are marked by an asterisk (adjusted p-value < 0.05; ANCOVA followed by contrast test, see methods). N, non-hair; H, hair cells.

Next, we performed 3D time-lapse imaging of WT, WT after the addition of BL and *bri1* meristematic epidermal cells at 3-h intervals for 13 h, 12 h and 10.5 h, respectively. We focus the analysis on epidermal cells because image acquisition of the inner tissues under 3D-optimized scanning conditions resulted in a lower signal-to-noise ratio. Due to the slow rate of cell displacement in the meristem, the changes made in growth directionality were quantified after approximately 12 h (Fig. 3b, Extended Data Fig. 4, Supplementary Videos 3 and 4, and Supplementary Table 6). When following the cells over time, we observed that fewer epidermal cells in the longitudinal axis divided in both *bri1* and BL-treated roots than in the WT (Extended Data Fig. 3a,b). No epidermal divisions occurred in other directions. Importantly, while BL dramatically increased the growth rate in the longitudinal axis, it had a moderate impact on growth rate in the radial axis, implicated in a lower growth rate of cell width. Consequently, the change in volume was only slightly higher than in the WT (Fig. 3c and Extended Data Fig. 3c). BL thus directs longitudinal growth at the expense of radial growth. The growth rate of cell depth was similar to that measured in WT cells (Fig. 3c and Extended Data Fig. 4c,d), suggesting that the lower depth upon BL treatment (Fig. 2h, H cells) was primarily the outcome of their short stay in the meristem (Extended Data Fig. 4c,d). In the absence of BRI1, epidermal cells grew significantly more slowly than the WT in their longitudinal and width directions and had either higher growth rates than the WT in the depth direction (non-hair cells) or the same growth rates as the WT (hair cells, Fig. 3c,d, Extended Data Fig. 4c,d and Supplementary Table 5). Thus, *bri1* cells are larger in depth not only due to the longer time spent in the meristem but also because of their faster growth rate in this direction. Taken together, meristematic cells with high and low BR signalling have distinct growth rates in opposing directions, which occurs over a distinct duration. The overall differences in growth rates and their directionalities were implicated in a lower rate of anisotropy growth in *bri1* cells and a higher rate after 12 h of root exposure to BL, compared with the WT (Fig. 3e). The corresponding BL-treated cells, however, had a relatively smaller difference in volume growth rate than the WT. Together, these kinematics and 4D analyses established BR signalling as setting the dynamics of the directionality of growth, where a given axis grows at the expense of the other (Fig. 3c–e and Extended Data Fig. 4c,d).

## Cell length is inversely correlated with radial growth of the meristem

**Fig. 4:**
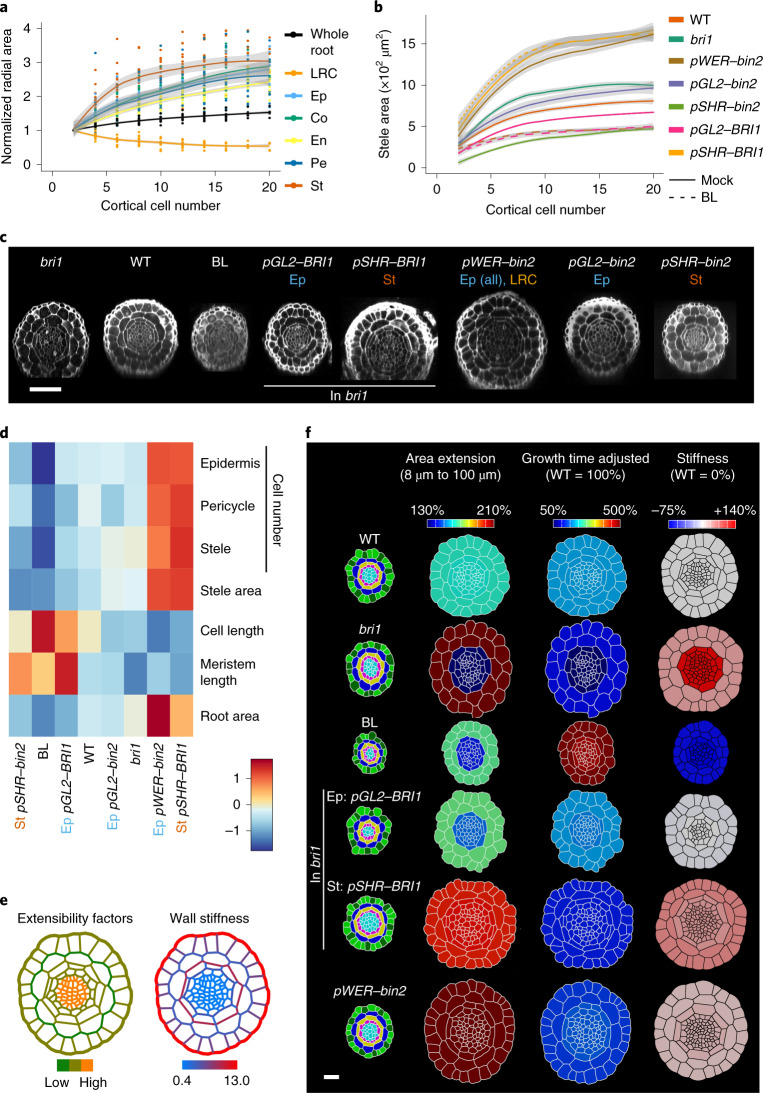
BR signalling links cell geometry and tissue-specific mechanical properties to radial meristem growth. **a**, Relative change in radial area of the root meristem and its constituent tissues along the meristem, as indicated by the position of cortical cells, normalized to the value at the QC. Ep, epidermis; St, stele. *n* = 24 roots. **b**, Stele area. The numbers of individual roots used in the experiment are 22, 9, 14, 9, 5, 11, 5, 6 and 6 for WT, *bri1*, WT + BL, *pGL2–BRI1*, *pGL2–BRI1* + BL, *pSHR–BRI1*, *pWER–bin2*, *pGL2–bin2* and *pSHR–bin2* (the statistics are shown in Supplementary Table 5). The shading indicates standard error bounds as calculated by loess. **c**, Confocal images showing radial sections of WT, *bri1*, WT treated with BL and lines with tissue-specific expression of BRI1 and *bin2* as in **b**, taken 100 μm from the QC. Scale bar, 50 μm. **d**, Heat map representing the mean meristematic parameters of the radial axis (root area at 100 μm, stele area and number of cells in the epidermis, pericycle and stele) and of the longitudinal axis (average cortical cell length and meristem length) of the lines in **b**. 6 ≤ *n* ≤ 24 roots. Note the inverse correlation between longitudinal and radial parameters across lines. **e**, Simulation model for radial growth in the WT root, showing the relative distributions of extensibility factors (left) and wall stiffness (right) across cell walls. Note the stiffer outer epidermal and outer endodermal walls, forming the two radial rings. **f**, Quantification and modelling of the radial growth in WT, *bri1*, BL, BRI1 limited to the epidermis (*pGL2–BRI1*) and stele (*pSHR–BRI1*) in the *bri1* background, and *pWER–bin2*. Cross sections of the initial templates at 8 μm from the QC, coloured by cell type and tissue (left), were compared with their corresponding 2D segmentations at 100 μm and the relative area extension in the outer and inner tissues (second from left, ‘Area extension’). The third column (second from right, ‘Growth time adjusted’) shows the incorporation of both time and area extension required for cells to displace from 8 μm to 100 μm (relative to the WT). The fourth column (right, ‘Stiffness’) depicts the cross section obtained from simulations. It shows the relative change in stiffness, compared with the WT, in the inner and outer tissues (see Supplementary Table 9 for the numeric data for the heat map). 3 ≤ *n* ≤ 8 roots.

**Extended Data Fig. 5:**
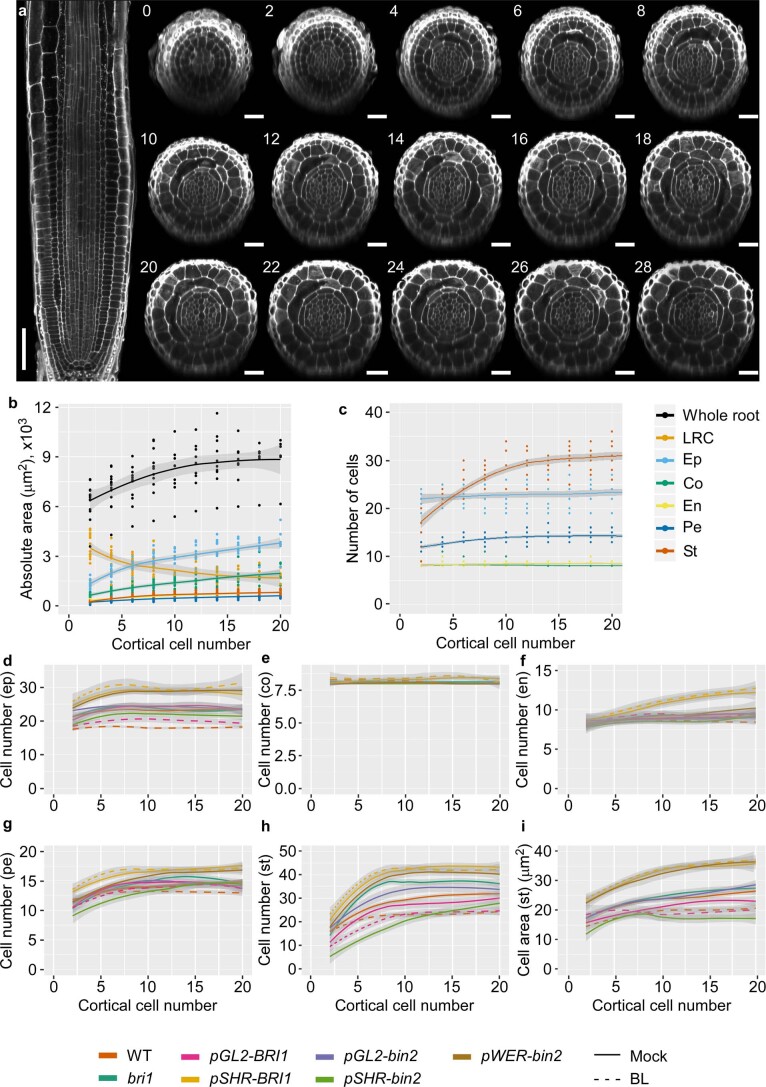
Quantification of growth parameters in the radial axis and their modulation by BR signaling. **a**, Confocal images of a WT root and corresponding sequential radial section taken at the QC, marked by the position of the cortical cell (2 closest to the QC). Longitudinal scale bar = 50 μm, radial scale bar = 20 μm. **b**, Radial WT root area, as measured from radial cross-sections of the different tissues composing the root at a given distance from the QC, marked by position of cortical cells. n = 24 roots. **c**, Number of cells in radial cross-sections of the different tissues composing the WT root, at a given distance from the QC, marked by position of cortical cells. n = 24 roots. **d-h**, Number of cells in radial cross-sections of the different tissues composing the root, at a given distance from the QC, marked by position of cortical cells in lines and number of individual roots, as in Fig. 4b. **i**, Average area of stele cells of lines as in **d-h**. Shades are standard error bounds as calculated by loess. LRC, lateral root cap; ep, epidermis; co, cortex; en, endodermis; pe, pericycle; st, stele.

Moving from the cell to the whole-organ level, we next asked if BL modulation of cell geometry is correlated with radial growth of the meristem. To address this question, radial growth of the different tissues along the meristem was quantified as a function of distance from the QC (as determined by the position of cortical cells along this distance) (Fig. 4a and Extended Data Fig. 5a–c). While the LRC area decreased with distance from the QC, all other tissues grew radially, most notably in the stele, with higher growth occurring at a distance corresponding to cortical cells 6–8 from the QC (Fig. 4a). This was associated with increased stele and pericycle cell numbers, after which, cell divisions gradually decreased and stopped around the positioning of cortical cell 20 from the QC (Extended Data Fig. 5c).

Next, we quantified the effect of BR signalling on radial growth and assessed the correlation between this parameter and the BR effect on cell length (as a simple derived parameter of cell geometry) and meristem length. To test whether these correlations are affected or maintained by tissue-specific perturbation of BR signalling, we similarly analysed lines with tissue-specific activation or inhibition of BR signalling (Fig. 4b,c and Extended Data Fig. 5d–i). These included lines with BRI1 targeted to the epidermis and stele tissues in the *bri1* background using the *pGL2* (directing expression to non-hair cells) and *pSHR* promoters, respectively^16^. WT lines with *bin2-1* (a dominant version of BIN2, hereafter *bin2*) driven by the same promoters and by *pWER* (which directs expression to epidermal cells and LRC) were used to inhibit BR signalling in these tissues^19^. As expected, WT treated with BL had a narrow stele, while *bri1* was significantly wider than the WT (Fig. 4b–d). Epidermal BRI1 activity was sufficient to limit the stele area of *bri1* in accordance with ref. ^17^, and this *pGL2–BRI1* line was not significantly different from the WT. Moreover, radial growth was further restricted in response to BL in this line, similar to WT treated with BL, supporting the epidermal control of meristem size^16,18^ (Fig. 4b–d and Extended Data Fig. 4d–i). By contrast, the expression of BRI1 in the stele greatly enhanced its size and did not respond to the addition of BL. The expression of *bin2* in the stele (*pSHR–bin2*) resulted in a smaller stele area than the WT, and *bin2* expression in the outer tissues (*pGL2–bin2*) had a stele area similar to that of *bri1* or wider than *bri1* (*pWER–bin2*), as was seen when BRI1 was limited to the stele (Fig. 4b,c and Extended Data Fig. 4d–i). Together, BR signalling in the outer and inner tissues had opposing effects on radial growth (negative and positive, respectively). When comparing all lines and treatments, an overall inverse correlation between the size of the radial axis (radial area and cell number) and that of the longitudinal axis (average cortical meristematic cell length and meristem length) was observed (Fig. 4d). BR modulation of cell geometry thus correlates with radial growth of the meristem.

## Simulation model of radial growth and its control by BR signalling

**Extended Data Fig. 6:**
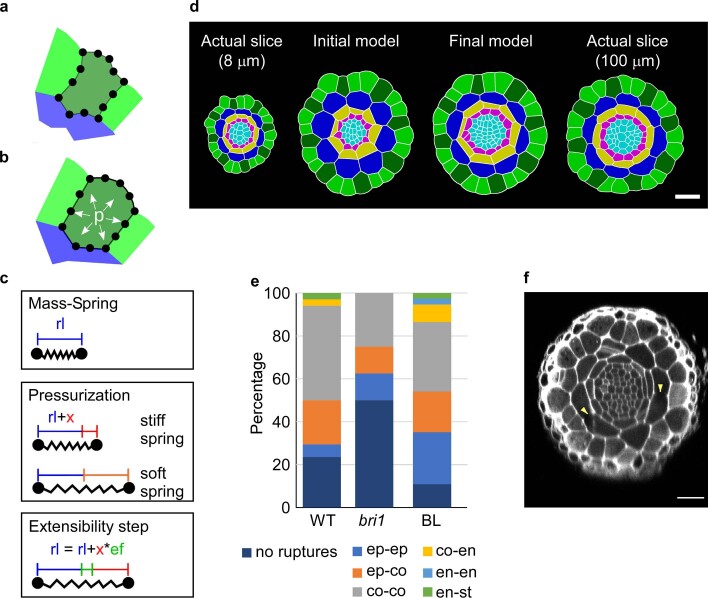
Mechanical model of tissue-specific BR effects on radial growth of the meristem. **a**, Portion of a segmented mesh used as a model template showing a few cells of the epidermis and the cortex. The cells are colored by cell type: green is epidermis (light green: non-hair cells, dark green: hair cells), blue is cortex. Cell walls are represented by springs that are connected to junction points. **b-c**, A spring has a rest length (rl, in blue) defined by the template’s initial geometry. Turgor pressure (p) in the cells puts the springs under tension (**b**) and leads to elastic expansion (x, in red) of their length (**c**). Springs can differ in their stiffness. Here the spring on the outside epidermal wall (in red) is stiffer and expands less than the inner epidermal wall (in orange). Application of extensibility factors (ef) transforms a portion of the expansion (x) into an increase of the spring’s rest length (green color). **d**, Simulation of radial growth requires differential cell wall stiffness. Representative 2D segmentation of a radial optical slice 8 μm from the QC (actual slice 8 μm, left). Simulation model of radial root growth using uniform (second from left, initial model) and differential (second from right, final model) cell wall stiffness. The resulting model output was compared to 2D segmentation of an optical cross-section at 100 μm from the QC (non-virtual slice 100 μm, right). **e**, Quantification of cell wall damage (ruptures) in WT, *bri1* and WT treated with BL during sample preparation. Number of roots used are 23,7,18 for WT, *bri1*, and BL respectively. Note that the epidermis and endodermis are less affected in all samples. *bri1* roots have the fewest cell wall ruptures while BL-treated roots have the most. Scale bars = 20 μm.

To understand how BR signalling controls growth, we modelled growth in the radial direction with a mechanical model of a 2D cross section of the root in the meristem (Extended Data Fig. 6 and Supplementary Information). To explore a model with minimal assumptions, we initially assigned uniform stiffness and extensibility factors to all cells in a cross section of the WT root at 8 µm and ran the growth simulation until the total area matched that of the corresponding 100 µm section (Extended Data Fig. 6d and Supplementary Video 5). Analysis of the tissue-specific areal change found large discrepancies. Cells of the outer tissues (epidermis and cortex) grew much more in the simulation (compared with the actual, non-virtual 100 µm section) and ended up 12% too large, whereas cells of the inner layers (endodermis and stele) grew less, with their final area 31% too small. This suggests that different tissues must have different effects on regulating the mechanical parameters that control growth.

To selectively modulate the mechanical parameters of the tissues, we relied on previously reported studies and on observations in this study. For example, it is often thought that the epidermis plays a major role in controlling growth^29^. Some authors liken plant tissue to a balloon, with the outer epidermal wall restricting the growth of the cells within^30,31^. The outer epidermis is also considerably thicker in many plant organs^29^, including the root meristem^32^. There is also indirect evidence that the endodermal layer of cells may have a prominent role in controlling growth. The flat shape of these cells gives the impression that they are constraining the stele, and among all tissues analysed, the shape of the endodermis was less affected as a function of distance from the QC (Extended Data Fig. 3). We also found that when handling samples for imaging, occasional ruptures of some of the cells occurred (Extended Data Fig. 6e,f). Quantification of these events revealed that the outer epidermal cell wall fully resisted this damage and that the endodermal walls were less affected than other cells in the WT, *bri1* and WT treated with BL (Extended Data Fig. 6e). Interestingly, a larger proportion of *bri1* roots maintained intact cell walls compared with the WT, while a lower proportion of WT treated with BL maintained intact walls, possibly reflecting distinct cell wall properties controlled by BR signalling. We therefore stiffened the epidermal walls and the outer and inner endodermal walls in our model and lowered the stiffness of the innermost walls in the pericycle and stele (Supplementary Table 7, Fig. 4e and Extended Data Fig. 6d). Finally, we adjusted the extensibility factors to achieve a good fit for the WT sample (Supplementary Table 7) and achieved a close match (within 0.1%) to the growth for both the inner and outer tissues (Fig. 4e, Extended Data Fig. 6d and Supplementary Table 7). Overall, our model can be viewed as a dual-ring structure, with the stiffer epidermal and endodermal walls having a dominant role in regulating growth.

Having established a model for radial growth that involves tissue-specific constraints, we asked to what extent these parameters could reproduce the *bri1* mutant phenotype. With WT parameters, we found that the inner tissue area grew too much (+18%), whereas the outer tissue area grew too little (−6%). Moreover, the model required 6% more time steps than the WT to grow to the size of the 100 µm slice, whereas *bri1* requires 76% more actual time (Fig. 3a and Supplementary Table 9)—that is, the model also considers hereafter a change in the rate of cell displacement relative to the WT (Supplementary Table 3). We were able to fit the model to the observed growth in *bri1* by increasing the stiffness of the cell walls of the inner tissues by 138% and the outer by 42% when matching the actual time (Fig. 4f, Supplementary Table 8 and Supplementary Video 6). Thus, although the *bri1* meristem is wider than the WT, it grows more slowly in the radial direction but for a much longer time. The simulation supports the idea that BRI1 has a tissue-specific effect on radial growth, the loss of which causes the cell walls of the inner tissues to be considerably more affected than the outer cell walls.

Adding BRI1 expression back into the stele in *bri1* (*pSHR–BRI1*) counterintuitively leads to an even wider root with exaggerated radial growth, beyond that of *bri1* (Fig. 4 and Extended Data Fig. 5). In our model, a good fit was achieved (considering calculated relative time to WT, Supplementary Table 3) with an increase in stiffness in the outer tissues of 41% over the WT and an increase in stiffness in the inner tissues of 32% (Supplementary Table 8, Supplementary Video 6 and Fig. 4e). This corresponds to a 45% decrease in stiffness in the inner layers compared with *bri1* and a 1% difference in stiffness in the outer layers compared with *bri1*. Thus, although the phenotype looks to be farther away from the WT, in the model it is a rescue of the radial growth specifically in inner tissues. The *pWER–bin2* line shows a similar phenotype and can be fit with a 47% decrease in stiffness in the inner layers compared with *bri1* and a 16% decrease in the outer.

By contrast, when BRI1 was restored to the epidermis in *bri1* (*pGL2–BRI1*), the simulation matched the actual growth when the WT stiffness was used for the inner tissues and the outer tissues were softened by only 3% (Fig. 4f, Supplementary Table 8 and Supplementary Video 6). It thus appears as a slight over-rescue in the radial growth of outer tissues but also a complete rescue of the inner tissues, in agreement with the rescue of *bri1* radial and longitudinal parameters (Fig. 4d). Indeed, the longitudinal growth rate of *pGL2–BRI1* meristematic cells differed from a complete WT rescue by only 3.7% (Supplementary Table 8). This suggests that BRI1 activity in the epidermis rescues radial and longitudinal growth in both the inner and outer tissues.

Finally, we simulated radial growth upon BL treatment of a WT root adjusting for the actual time the cells spend in the meristem (Fig. 3a, Supplementary Table 8 and Supplementary Video 6). A match was found in the simulation by reducing the stiffness of the inner layers by 75.5% and the outer layers by 71.5% (Fig. 4f). This suggests that BL treatment elicits a similar effect in both the inner and outer layers, which need to be softened by a similar amount.

## Discussion

Understanding morphogenesis control requires a multiscale analysis of the factors involved. Using the root meristem as the model organ and BR signalling as one of these factors, our work established the key role of BR as a controller of cellular growth directionality from the onset of cell production, while cell volume was still stable. We then linked geometry at the cellular scale to radial meristem growth, and we propose a model in which BR signalling controls radial growth via interaction with tissue-specific mechanical constraints.

A long-standing hallmark of BR signalling, as concluded from 2D studies, is the promotion of cell elongation in different developmental and physiological contexts^33^. For example, kinematics analyses of the elongation zone of the root showed that *bri1* cells reach a lower maximal growth rate that ceases early^16^. Unlike the rapid elongation of cells in the elongation zone, the growth of meristematic cells is very slow, and thus direct kinematics measurements are limited and require higher spatial resolution^34^. The approach taken here was to generate precise 3D geometry datasets for thousands of meristematic cells across tissues and treatments. These data included time-lapse imaging for the quantification of volumetric growth rates in 3D of single cells. This analysis revealed that BR signalling increases cell anisotropy by controlling growth rates in different directions, with a relatively minor effect on cell volume. In response to high BL levels, meristematic cells greatly accelerate their elongation rate while slightly decelerating growth rate in width, with the rate of volume increase along the meristem remaining closer to the untreated control. When comparing cell shape after long-term exposure to BL, as in our 3D analysis, a lower width becomes significant when comparing with *bri1*. In the absence of BR signalling, growth rates were lower than in the WT, except non-hair cells that grew faster in depth. These findings align with a compensatory process acting on cell geometry, with volume being a primary geometric constraint. The incorporation of time also established *bri1*’s slow rate of cell expansion and associated long cell cycle duration^13,16^. We also demonstrate that these cells spend more time in the meristem and thus have higher total radial growth, while the opposite occurs when BR signalling is high. Recent studies in the shoot apical meristem suggested that the overall stability of meristematic cell volume results from feedback between cell cycle and growth^35^. However, in the root meristem, cell volume gradually increases as cells are displaced from the QC. A computational model proposed that root cells sense their length and stop elongating when reaching a threshold value, depending on BRI1 acting in the meristem^36^. While several hypotheses are valid (for example, the mechanical strength of the cell scales with volume and limits cell size^37^), our data suggest that cells sense a threshold volume and that the target volume increases with distance from the QC. It is plausible that adjusting growth rate in the radial direction, as is achieved by differential BR signalling intensities, is a means of stabilizing a coherent distribution of cell volume. Alternatively, growth directionality can be modulated when volume is a primary geometric constraint.

BR signalling can control directional growth by modulating microtubule arrangement^38–42^, which in turn guides the positioning of cellulose microfibrils. In the root meristem, a transverse (perpendicular to the root axis) orientation dominates, except under the outer epidermal wall^43^. The arrangement of the latter, however, becomes transverse in the presence of high BR signalling, as in cells leaving the meristem^42^, suggesting that BR signalling could modulate the anisotropy of the cell wall, which also guides cell shape^44–46^. Earlier experiments using stem segments revealed that BR promotes wall loosening^47,48^, in a process involving the alteration of its mechanical properties^47^, which was recently supported^49^. When a load was applied to stem segments, a higher frequency of breakage was observed in the BL-treated samples, indicating mechanical weakening^48^. Here, we also observed an increased frequency of ruptures when handling root samples treated with BL. In all treatments, these ruptures tended to occur in specific walls, in agreement with differential mechanical properties between tissues. Indeed, differences in growth control among tissues were proposed to be part of root elongation^50^.

To understand how the different tissues interact to control growth, we developed a mechanical model of a growing cross section of the root. In the longitudinal direction, the growth rates of all cell layers must be similar. Radial growth, however, can involve differential growth rates among tissues. The results from the model suggest that the mechanical properties of the different tissues of the root meristem are not uniform, and that the inner and outer tissues must have differential regulation of growth. The model shows that uniform stiffness and extensibility factors throughout the root meristem are not sufficient to explain the cellular patterning observed in vivo. We found that a dual-ring structure, with stiffer epidermal and endodermal cell layers, presents a simple physical arrangement that can regulate growth. Since plant cell growth is thought to be a stress relaxation process, stiffer cell layers would have a dominant role in controlling the growth of the softer layers below them. After fitting the dual-ring model to WT growth rates, we explored how BR signalling can regulate growth in the model. The short and wide meristem phenotype of *bri1* can be interpreted as a trade-off between longitudinal and radial growth, as also observed upon tissue-specific perturbations of BR signalling and as supported by our finding that cell volume is relatively unchanged. However, when fitting the model to the observed duration of growth, it becomes clear that the radial growth is reduced as well, albeit less than in the longitudinal direction. We also found that to fit tissue-specific growth, the walls in the inner tissue had to be stiffened more than those in the outer tissues. The model thus suggests that BR signalling has a differential effect on the inner and outer tissues. When BRI1 is expressed in the stele in the *bri1* background (*pSHR–BRI1*), the meristematic cell length and meristem length are not changed; however, the meristem becomes wider. The initial phenotype seems counterintuitive, as it is opposite to the direction of rescue. However, when timing is taken into account in the model, we found that *pSHR–BRI1* can be fit by restoring the parameters to almost match those of the WT in the inner tissues, largely rescuing the radial growth there. Yet, the largely unaffected longitudinal growth in *pSHR–BRI1* reinforces the notion that BR signalling in the stele mostly promotes radial growth. This is in contrast to expressing BRI1 in the epidermis only, where both the radial and longitudinal growth are largely restored in all tissues. In agreement with this result, reducing BR signalling strength in the outer tissues (as in *pWER–bin2*) yielded inner versus outer values that favour radial growth, similar to *SHR–BRI1*. BR signalling thus differentially affects the inner and outer rings, with the activity in the outer layer playing a more dominant role in controlling and promoting longitudinal growth. Together, our experimental data and simulation propose a plausible model for how tissue-specific decoding of BR signalling at the cell level drives mechanical changes. This probably controls growth anisotropy and shapes the root meristem.

## Methods

### Plant material, growth conditions and chemical treatments

All *Arabidopsis* (*Arabidopsis thaliana*) lines were in the Columbia-0 (Col-0) background. The following lines were used: *pGL2–BRI1* and *pSHR–BRI1*^16^, 35S–eGFP–Lti6b^51^, *pWER–bin2–NeonGreen* (line 24) and *pSHR–bin2–GFP*^19^, *pGL2*–*bin2*–GFP (this study), and *bri1-116*. Seeds were sterilized and germinated on one-half-strength Murashige and Skoog (MS) medium supplemented with 0.8% plant agar, 0.46 g l^−1^ MES (pH 5.8) and 0.2% (w/v) sucrose. Plates with sterilized seeds were stratified in the dark for two days at 4 °C and then transferred to 22 °C and to a 16 h light/8 h dark cycle (70 μmol m^−2^ s^−1^) for seven days. For BL treatment, three-day-old seedlings were transferred to 3 nM BL. BL activity of the used batch was equivalent to 0.1 nM, corresponding to the activity in our earlier BL batch^52^.

### Confocal microscopy

For snapshots of live roots, fluorescent signals were detected using an LSM 510 META confocal laser-scanning microscope (Zeiss) with a ×25 water immersion objective lens (NA 0.8). The roots were imaged in water supplemented with 10 mg ml^−1^ propidium iodide (PI). PI and eGFP were viewed at excitation wavelengths of 561 nm and 980 nm TiSapphier multi-photon, respectively. Fluorescence emission was detected at 575 nm for PI and with a 500-nm and 530-nm bandpass filter for eGFP. For live imaging (Fig. 3a and Supplementary Video 2), *35S–eGFP–Lti6b* in the WT and *bri1-116* backgrounds were imaged using optical plates (Ibidi, 35-mm dish) and an LSM 710 inverted confocal laser-scanning microscope (Zeiss) with a ×20 air objective lens (NA 0.8). eGFP was viewed with an excitation wavelength of 488 nm, and emission was detected using a 500-nm and 530-nm bandpass filter. To prevent the roots from drifting, channels with a 160-µm radius were moulded using acupuncture needles inside MS media supplemented with 2% bacto agar. The seedlings were germinated on 0.5 MS plates and were positioned inside the channels when they were seven days old and then flipped over to fit inside the optical plates. In cases of chemical or hormonal treatment, the treatment was added to the bacto-agar-supplemented MS during preparation.

For 3D segmentation, the roots were fixed using the mPS-PI protocol^53^ and placed in a chamber made of 200-μm-thick dual-sided tape (to prevent the sample from changing shape due to cover slip pressure). The tape was glued to a slide, the roots were placed inside and a cover slip was glued above. Imaging was conducted using an LSM 510 META confocal laser-scanning microscope with a ×40 oil immersion objective lens (NA 1.3). PI was viewed with an excitation wavelength of 561 nm, and emission was collected with a 575-nm bandpass filter, using a fine pinhole of 1 μm and *Z*-steps of 0.8 μM. For 4D image acquisition, moulds with channels as described above were made to fit a microscope slide. The roots of seven-day-old seedlings were positioned in the channels, closed with a cover slip and kept vertically in the growth chamber between imaging sessions. Imaging was performed every three hours.

### Segmentation analysis

All acquired images underwent pre-segmentation processing, including background subtraction and signal enhancement, using Fiji^54^. MorphoGraphX^28^ (version 2.0) was used for all segmentations (that is, 2D, 3D and 4D), followed by manual corrections. The images were segmented using the Insight Toolkit (https://itk.org) morphological watershed processes in MorphoGraphX, and a volumetric (3D) mesh was extracted. The 3D meshes were analysed using the 3D Cell Atlas pipeline^55^, which uses an organ-centric coordinate system to assign directions to each cell. Cell sizes (length, width and depth) were obtained by measuring the size of each cell along these directions through the cell’s centre of gravity. Cell geometry data were exported to CSV files and further analysed in R version 4.0.2^56^. For 4D analysis, lineage tracking was used to track a given cell between time points. For rate calculations, geometrical parameters (cell length, width, depth and volume) were extracted, and their difference between the beginning and end of the video was calculated and divided by the duration of the video (hours). If a given cell was divided, the length and volume of the daughter cells were summed, while their depth and width values were averaged. Cell divisions were extracted on the basis of lineage assignment. The 4D videos were generated using Abrosoft FantaMorph version 5.0 (https://www.fantamorph.com) software using the first and the last time points of segmentations and lineage representation.

### Radial analysis

Fixed roots were imaged with the microscope settings used for the 3D analysis and loaded into Fiji. The roots were straightened, and a horizontal line was drawn at the point of the intended slicing. The dynamic reslice Fiji tool was used to generate an optical cross section. A heat map (Fig. 3d) was produced using R with the heatmap.plus package. For each line, the radial mean was calculated around the positioning of cortical cell 20 from the QC and was scaled (normalized) using the intrinsic heatmap.plus function.

### Classification of meristematic cells

Cells were classified into meristem and elongation zone cells as follows. Using the expectation maximization algorithm as implemented in the mixtools R package^57^, a two-Gaussian mixture model was fitted to the cell length parameter in each combination of tissue and BR condition (WT and each of the BR signalling perturbations) in roots. The probability of being in the short-length Gaussian was calculated for each cell. Cells with a probability >0.8 were considered meristem cells. Further analyses were performed on the meristematic cells.

### Statistical analysis and quantification of the BR signalling contribution

For the 3D data, the experimental design had fixed (that is, BR signalling perturbations and distance from the QC) and random (that is, biological replicate) factors. Hypothesis testing was therefore performed with mixed-model ANOVA using the R lmer function^58^. ANOVA assumes a normal distribution. We therefore visualized the data and, if required, transformed the parameters to achieve proxies for normal distributions. The transformations are detailed in Supplementary Table 2. Post-hoc tests were performed using the Tukey procedure, as implemented in the multcomp package^59^, in each model that was significant after correction for multiple hypotheses. Correction for multiple hypotheses was performed using a two-step adaptive procedure^60^. Briefly, in the first stage, we used the Benjamini–Hochberg procedure with *α* = 0.05. This stage resulted in 17 significant models of the 25 possible models. In the second stage, we performed the Benjamini–Hochberg procedure on the post-hoc comparisons with *α* = 0.05 × 17/25. This procedure guarantees that the false discovery rate is kept at 0.05 throughout the entire experiment. To determine the percentage of variation explained by BR, we first used the Insight package^61^ to calculate the variance explained by the fixed factors, the random factors and the residual variance. Then, to isolate the variance explained by different BR signalling, we multiplied the fixed variance by the proportion of the sum of squares of the BR conditions.

The 2D data (stele area) were analysed using a mixed-model ANOVA, as described for the 3D data. Since we were interested only in a small part of all possible genotype comparisons, we generated a model for each pair of interest. As a result, no post-hoc test was required. Correction for multiple hypotheses was performed using the Benjamini–Hochberg procedure with *α* = 0.05.

For the 4D data, each cell was measured at two time points. We selected cells located up to 60 μm from the QC at the first time point and up to 100 μm at the second time point. This ensured that the cells in the comparison were residing in the meristem throughout the experiment. ANCOVA was performed using the lm function^62^ for each geometrical parameter, with treatment and distance from the QC as the main factors. Each ANCOVA was started with an interaction model, which was then simplified when possible, as described in *The R Book*^63^. Post-hoc comparisons between treatments were performed using the contrast package^64^.

### Kinematics analysis

To directly quantify the cell displacement rate, roots were positioned in channels on an optical plate (as previously described) and imaged at 30-min intervals over 6 h. The resulting images were subjected to stitching and regression correction to overcome 3D drifts of the sample (all within Fiji). The same cell was traced, and its distance from the QC at *t* = 0 h and *t* = 6 h was recorded. The measurements were plotted, and the slope was derived from the regression analysis using a linear fitted curve (*R*^2^ > 0.9) to obtain the change in the rate of meristematic cell displacement up to 100 μm from the QC, which was presented as a value relative to the WT and the time a cell spends between 8 μm and 100 μm from the QC (Supplementary Table 3).

To calculate the relative change in the rate of cell displacement to the WT for modelling, *bri1*, *pGL2–BRI1*, *pSHR–BRI1* and *pWER–bin2* cell production rates were evaluated on days 6 to 7. This was performed by dividing the length of the root measured between days 6 and 7 by the average mature cell length. Next, the average meristematic cell length was determined and was used to calculate the total length that left the meristem in this time period (that is, the average meristematic cell length times the cell production rate). The meristem length was calculated as the meristem cell number times the average meristem cell length and was applied to calculate the change in cell displacement rate (the meristem length divided by the length that left the meristem), relative to the WT. A good match was found between the calculated ratio of change in the cell displacement rate of *bri1* versus the WT and the ratio derived directly from live imaging (compare the two right columns in Supplementary Table 3), supporting our calculation method for the genotypes that were not subjected to a direct kinematics analysis.

Since our regression analysis from the live-imaging-based quantification showed a linear increase in displacement rate, we also calculated the linear displacement rate increase function between the origin of axes and the end of the meristem. On the basis of this function, the duration of cell displacement along the meristem was calculated for the WT, *bri1* and the WT treated with BL (Supplementary Table 4).

### Model

The mass-spring model of the root cross section was written in C++ using the MorphoDynamX (www.MorphoDynamX.org, version 2.0) simulator in the VLab modelling framework^28,65^. See the Supplementary Information for further details.

### Reporting Summary

Further information on research design is available in the Nature Research Reporting Summary linked to this article.

## Supplementary information

Supplementary Information Description of the model.

Reporting Summary

Supplementary Table 1 3D geometry parameters of WT (col), *bri1* and BL (colBL).

Supplementary Tables 2–5 and 7–9 Tables 2–5 and 7–9.

Supplementary Table 6 4D geometry parameters of WT (col), *bri1* and BL (colBL).

Supplementary Video 1 Root meristem morphology and segmentation. Animation of the WT root meristem. Shown is the raw confocal stack with the cell wall signal (greyscale) and the 3D cellular segmentation in arbitrary cell colours and in cell type colours. Green indicates hair (dark green) and non-hair cells of the epidermis, blue indicates cortex, yellow indicates endodermis, pink indicates pericycle and cyan indicates stele.

Supplementary Video 2 Growth of WT, *bri1* and BL-treated roots. Root growth of WT, *bri1* and BL-treated roots, harbouring 35S–Lti6b–eGFP. The roots were imaged every 30 minutes for a total duration of 6 hours. The videos were acquired using optical channels under an inverted confocal microscope. STD *Z*-projection is presented. These videos were used to measure meristematic cell displacement (Fig. 3a).

Supplementary Video 3 3D segmentation over time. WT, *bri1* and BL-treated roots were imaged for 13, 10.5 and 12 hours, respectively. The images were segmented, and the initial and final images were morphed to represent root growth (see also Fig. 3 and Extended Data Fig. 4). Elongation and meristem zones are shown.

Supplementary Video 4 3D segmentation of meristematic cells over time. As in Supplementary Video 3, showing the meristematic zone and single meristematic cells.

Supplementary Video 5 Model of WT radial growth using equal wall stiffness. A representative WT root was used to model radial growth from a section located at 8 μm from the QC to 100 μm from the QC. A uniform stiffness and extensibility factor were assigned to all the cells, resulting in deviation from the actual shape of the cross section (Extended Data Fig. 6).

Supplementary Video 6 Simulation of BR signalling control of radial meristem growth. Radial growth simulation of WT, *bri1*, BL-treated roots, *pGL2–BRI1*, *pSHR–BRI1* and *pWER–bin2* (see also Fig. 4).

## Data Availability

The data supporting the findings of this study are available within the paper and its Supplementary Information files. Lists of 3D parameters are made available as Supplementary Table 1. Lists of 4D parameters are made available as Supplementary Table 6.

## Code availability

The code used in this study for statistical analysis is available on GitHub at https://github.com/BKU-Technion/BR_control, and the code for modelling is available on GitLab at https://gitlab.mpcdf.mpg.de/sstrauss/root-model-fridman-et-al-2021.
