# Author Correction: The root meristem is shaped by brassinosteroid control of cell geometry

**DOI:** 10.1038/s41477-022-01095-0

**Published:** 2022-01-11

**Authors:** Y. Fridman, S. Strauss, G. Horev, M. Ackerman-Lavert, A. Reiner-Benaim, B. Lane, R. S. Smith, S. Savaldi-Goldstein

**Affiliations:** 1 Faculty of Biology, Technion-Israel Institute of Technology, Haifa, Israel; 2 Department of Comparative Development and Genetics, Max Planck Institute for Plant Breeding Research, Cologne, Germany; 3 Lorey I. Lokey Interdisciplinary Center for Life Sciences and Engineering, Technion – Israel Institute of Technology, Haifa, Israel; 4 Clinical Epidemiology Unit, Rambam Health Care Campus, Haifa, Israel; 5 Department of Computational and Systems Biology, John Innes Centre, Norwich, UK

**Keywords:** Plant morphogenesis, Brassinosteroid, Root apical meristem

Correction to: *Nature Plants* 10.1038/s41477-021-01014-9, published online 15 November 2021.

In the version of this article initially published, there were errors in the Fig. 1 color key. In the key, “Cortex” should have been by the blue bar, rather than yellow, and “Endodermis” by the yellow bar, rather than blue. The errors have been corrected in the html and PDF versions of the article.

