# Supplementary material for: The root meristem is shaped by brassinosteroid control of cell geometry
Source: Nat Plants. 2021 Nov 15;7(11):1475–84. doi: 10.1038/s41477-021-01014-9 (PMC8592843; doi:10.1038/s41477-021-01014-9)
Supplement: Supplementary file 1 — Description of the model. [file 41477_2021_1014_MOESM1_ESM.pdf]

nature portfolio

https://doi.org/10.1038/s41477-021-01014-9

Supplementary information

The root meristem is shaped by brassinosteroid control of cell geometry

In the format provided by the authors and unedited

Supplementary description of the model

The simulations model of a growing root cross section

In the model, cell walls are represented as springs connected to vertices that represent the cell junctions (Extended Data Fig. 6a-c). The walls have a rest length that is initially taken from the starting template of a cross section of the root 8 µm from the QC (Extended Data Fig. 6a). Turgor pressure is applied only to the boundary, that is the outer epidermal walls, as it cancels out on inner walls. Pressure is applied by adding a force perpendicular to each boundary cell wall segment proportional to the segment length. This force is then added to the junction points of the boundary wall. These forces cause the boundary to move outwards, and the cell walls both on the boundary and the interior to stretch (Extended Data Fig. 6b). Growth is implemented by increasing the rest lengths of the springs in a strain relaxation process. The rest length extension during each growth step is calculated as the product of elastic strain multiplied by a wall extensibility factor that represents the action of cell wall remodeling gene products (Extended Data Fig. 6c). Note that each spring represents a pair of walls between cells except for the outer epidermal walls. Both the elasticity of the cell wall (stiffness of the springs) and the extensibility factors for each wall can be specified for different cell wall pairs by tissue type, for example, cortex-cortex, epidermis-cortex, epidermis-outside, etc. The strain relaxation growth model means that growth can be modulated by changing the elasticity (stiffness) of the walls or by changing the extensibility. Lowering the wall stiffness will cause the wall to stretch more under turgor pressure and increase the growth, as will an increase in the extensibility factor. Although we are modeling the effect of BR signaling on growth, it remains unknown whether BR signaling affects the cell wall stiffness, extensibility factors or a combination of the two [1,2]. Here we chose to fit the WT reference model to a simple combination of extensibility factors and stiffnesses while matching the WT growth. Although it is possible to fit the model with a single uniform extensibility factor in the different cell layers, some cells would need to be several hundred-times stiffer than others, likely an unrealistic scenario. The same occurs when fitting with a single stiffness value in all the layers. From our reference WT model, we modelled BR signaling changes as differences in stiffness, although other combinations involving differences in extensibility would also be possible.

Cell division

The only cells that divide in the simulations are the cells in the pericycle and stele, which divide at threshold areas based on their cell type, estimated from the data. Although there were differences in average cell size between the genotypes, the growth of the innermost tissue was dominated by the stiffer endodermis, therefore a single value for cell size was used for each tissue type. Since very few tangential divisions occur in the epidermis and endodermis and none in the cortex (Extended Data Fig. 5d,f), cell division was not modeled for these layers.

Representative sample templates

The Arabidopsis root meristem reached a relatively stable width at around 15-20 cells from the QC, which corresponds to a distance of 60-80 µm (Fig. 4, Extended Data Fig. 5). Thus, we used a cross-section at 8 µm from the QC to represent an early stage of development and another section at 100 µm from the QC to reflect a later stage. This covers approximately 2.57 days (61.86 hours) of WT root growth (calculated from Fig. 3a). Model simulations were done on radial cross-sections at 8 µm and 100 µm distances from the QC, that were extracted from 3D images using Fiji's "dynamic reslice" tool. The 2D cross-section images were loaded into MorphoGraphX and segmented, starting with a square mesh in the XY plane covering the entire cross-section. The signal from the 2D cross-section was projected onto the mesh with a triangle size slightly smaller than the pixel size of the original image. The meshes were then manually seeded and segmented using the watershed segmentation process. In a final step, the mesh walls were smoothed.

For the initial model with uniform material properties and growth rates, the representative cross-section sample was chosen as the closest to the mean value obtained for areal expansion from 8 µm to 100 µm and subsequently used for modeling. For the different genotypes and treatments (WT, *bri1*, BL*, pSHR-BRI1*, *pGL2-BRI1* and *pWER-bin2-1*) we segmented between 3 and 8 replicates, from which we chose one representative sample for each genotype based on their sum of relative mean square errors of the growth of the outer (epidermis, cortex) and inner (endodermis, pericycle, stele) tissues. The samples with the smallest error scores were selected as representative samples, with the exception of the *bri1* sample, where we chose the second-best sample, as the best sample showed an overall asymmetry in the epidermis due to fewer LRC layers in one side of the root at 8 µm.

The main parameters of the mass-spring model were spring stiffness, extensibility factors (both assigned to the wall edges, see Extended Data Fig 6a) and max cell area (assigned to cells). See Table S7 for an overview of the stiffness and extensibility factors of the model and Table S8 for changes required to model the treatments and genotypes. The maximum cell area of stele was 30 µm^2 and 70 µm^2 for the pericycle. Relative time of cell displacement (Supplementary Table 3) was incorporated as a parameter relative to WT for all modelled genotypes (Supplementary Table 7).

References:

1 Zurek, D. M., Rayle, D. L., McMorris, T. C. & Clouse, S. D. Investigation of gene expression, growth kinetics, and wall extensibility during brassinosteroid-regulated stem elongation. *Plant Physiol* **104**, 505-513 (1994).

2 Wang, T. W., Cosgrove, D. J. & Arteca, R. N. Brassinosteroid Stimulation of Hypocotyl Elongation and Wall Relaxation in Pakchoi (Brassica chinensis cv Lei-Choi). *Plant Physiol* **101**, 965-968 (1993).
