# Supplementary material for: The root meristem is shaped by brassinosteroid control of cell geometry
Source: Nat Plants. 2021 Nov 15;7(11):1475–84. doi: 10.1038/s41477-021-01014-9 (PMC8592843; doi:10.1038/s41477-021-01014-9)
Supplement: Supplementary file 2 — Reporting Summary [file 41477_2021_1014_MOESM2_ESM.pdf]

Corresponding author(s): Sigal Savaldi-Goldstein

Last updated by author(s): Sep 13, 2021

Reporting Summary

Nature Research wishes to improve the reproducibility of the work that we publish. This form provides structure for consistency and transparency in reporting. For further information on Nature Research policies, see our Editorial Policies and the Editorial Policy Checklist.

Statistics

For all statistical analyses, confirm that the following items are present in the figure legend, table legend, main text, or Methods section.

n/a	Confirmed	
☐	☒	The exact sample size (*n*) for each experimental group/condition, given as a discrete number and unit of measurement
☐	☒	A statement on whether measurements were taken from distinct samples or whether the same sample was measured repeatedly
☐	☒	The statistical test(s) used AND whether they are one- or two-sided<br>*Only common tests should be described solely by name; describe more complex techniques in the Methods section.*
☐	☒	A description of all covariates tested
☐	☒	A description of any assumptions or corrections, such as tests of normality and adjustment for multiple comparisons
☐	☒	A full description of the statistical parameters including central tendency (e.g. means) or other basic estimates (e.g. regression coefficient) AND variation (e.g. standard deviation) or associated estimates of uncertainty (e.g. confidence intervals)
☐	☒	For null hypothesis testing, the test statistic (e.g. F, t, r) with confidence intervals, effect sizes, degrees of freedom and P value noted<br>*Give P values as exact values whenever suitable.*
☒	☐	For Bayesian analysis, information on the choice of priors and Markov chain Monte Carlo settings
☒	☐	For hierarchical and complex designs, identification of the appropriate level for tests and full reporting of outcomes
☐	☒	Estimates of effect sizes (e.g. Cohen's *d*, Pearson's *r*), indicating how they were calculated

Our web collection on statistics for biologists contains articles on many of the points above.

Software and code

Policy information about availability of computer code

Data collection	Cell geometry parameters from acquired images were quantified using MorphoGraphX version 2.0 (morphographx.org). Modeling was performed using MorphoDynamX version 2.0 (www.MorphoDynamX.org).
Data analysis	For data analysis, we used in R version 4.0.2. The R packages used in this study are listed in the code available in GitHub in: https://github.com/BKU-Technion/BR_control<br>Code availability<br>The code used in this study for statistical analysis is available on GitHub:<br>https://github.com/BKU-Technion/BR_control and the code for modeling is available on GitLab: https://gitlab.mpcdf.mpg.de/sstrauss/root-model-fridman-et-al-2021<br>Abrosoft Fantamorph V5 was used for visualization of the 4D movie.<br>Data availability<br>Lists of 3D parameters are made available as Supplementary Table 1. Lists of 4D parameters are made available as Supplementary Table 6.

For manuscripts utilizing custom algorithms or software that are central to the research but not yet described in published literature, software must be made available to editors and reviewers. We strongly encourage code deposition in a community repository (e.g. GitHub). See the Nature Research guidelines for submitting code & software for further information.

Data

Policy information about availability of data

All manuscripts must include a data availability statement. This statement should provide the following information, where applicable:

- Accession codes, unique identifiers, or web links for publicly available datasets
- A list of figures that have associated raw data
- A description of any restrictions on data availability

Data and code will be available upon publication

Field-specific reporting

Please select the one below that is the best fit for your research. If you are not sure, read the appropriate sections before making your selection.

☒ Life sciences ☐ Behavioural & social sciences ☐ Ecological, evolutionary & environmental sciences

For a reference copy of the document with all sections, see nature.com/documents/nr-reporting-summary-flat.pdf

Life sciences study design

All studies must disclose on these points even when the disclosure is negative.

Sample size	To quantify the geometry (3D) of virtually all cells of the meristem in epidermis, cortex, endodermis and pericycle, the number of plants for each treatment is at least 3, with hundreds of cells carefully segmented and quantified in each root. For 4D analysis, the sample count is lower since it was requiring 3D segmentations over several time points, for each of the treatments. The differences in growth rates in length among treatments in 4D are supported by the live imaging performed in 2D. For all other analysis performed on 2D root images, the sample size was determined based on common empirical knowledge in similar scientific studies.
Data exclusions	No data was excluded
Replication	In 2D and 3D experiments, samples from at least 3 independent experiments were used, and for pwer-bin2, 2 independent experiments were used. Where appropriate, we used the mixed model ANOVA . In this statistical approach each independent replication is considered random, resulting in a high benchmark for finding significant differences that confirms replicability.
Randomization	For transgenic plants / mutants, experimental groups are determined according to their genotype. For hormone treatments, plants at the same developmental stage were allocated to experimental groups at random, as described in Methods.
Blinding	Quantification is performed algorithmically, therefore all the geometrical parameters are quantified similarly for the different parameters.

Reporting for specific materials, systems and methods

We require information from authors about some types of materials, experimental systems and methods used in many studies. Here, indicate whether each material, system or method listed is relevant to your study. If you are not sure if a list item applies to your research, read the appropriate section before selecting a response.

Materials & experimental systems

n/a	Involved in the study	
☒	☐	Antibodies
☒	☐	Eukaryotic cell lines
☒	☐	Palaeontology and archaeology
☒	☐	Animals and other organisms
☒	☐	Human research participants
☒	☐	Clinical data
☒	☐	Dual use research of concern

Methods

n/a	Involved in the study	
☒	☐	ChIP-seq
☒	☐	Flow cytometry
☒	☐	MRI-based neuroimaging
